# The Skin Affections Met with in Bright's Disease

**Published:** 1900-03-24

**Authors:** 


### THE SKIN AFFECTIONS MET WITH IN BRIGHT'S DISEASE.

At the last meeting of the Royal Medical and Chirurgical Society Dr. Hugh Thursfield read a paper on the skin affections met with in Bright's disease. He was able to show pretty conclusively that among cases of Bright's disease a considerable number of instances of various kinds of skin disease were met with, but whether these diseases were really set up by the coexisting malady seemed to be by no means certainly established. The classification of these cases which he proposed was as follows: (1) The affections which characterised the early stages of renal disease—pruritus, urticaria, and eczema. (2) Those which occurred in the final stage and in uræmic conditions—the universal erythematous, bullous, or desquamative eruptions. (3) Purpura and other hæmorrhagic eruptions. (4) Those affections which were only seen with marked œdema, and which were in all cases, he believed, due to a local infection of the skin, usually by some one or other of the pyogenic micrococci. His remarks were especially directed to eruptions of the second class, and he gave some interesting examples of exfoliative dermatitis occurring in conjunction with Bright's disease. As to the causation of all the different forms of skin affections in Bright's disease, putting on one side those due to the implantation of pyogenic micrococci on an œdematous and injured skin, the most probable explanation appeared to be that they were due to some special toxin or toxins circulating in the blood. Thus they came into close relationship with the rashes which occurred in septicæmia and in connection with certain drugs, and, as in these instances, the toxin most probably acted not directly, but through the agency of the vaso-motor and trophic nerves. In commenting on this paper, Dr. Radcliffe Crocker said that one might regard the question from two points of view, either looking on the renal disease as the cause of the eruption, or the skin lesion as the cause of the albuminuria. He could not say that he had found that any special character of skin disease necessarily suggested a renal origin. Certainly some cases of pityriasis rubra did occur with albuminuria, but he believed that albuminuria was more often a late sequel of the skin affection than *vice versa*. It was, he said, only by the complete investigation of all the organs in any case of skin disease that its renal origin could be discovered. Not only might any form of renal affection give rise to these skin symptoms, but many varied kinds of skin lesion might be associated with renal disease.

